# Supplementary material for: Therapeutic paradigm of dual targeting VEGF and PDGF for effectively treating FGF-2 off-target tumors
Source: Nat Commun. 2020 Jul 24;11:3704. doi: 10.1038/s41467-020-17525-6 (PMC7382445; doi:10.1038/s41467-020-17525-6)
Supplement: Supplementary file 1 — Supplementary Information [file 41467_2020_17525_MOESM1_ESM.pdf]

SUPPLEMENTARY INFORMATION

Therapeutic paradigm of dual targeting VEGF and PDGF for effectively treating FGF-2 off-target tumors

K. Hosaka et al.

Corresponding author:
Yihai Cao, M.D., Ph.D., Department of Microbiology, Tumor and Cell Biology, Karolinska Institutet, 171 77 Stockholm, Sweden. Tel: (+46)-8-5248 7596, Fax: (+46)-8-33 13 99, E-mail: yihai.cao@ki.se

Supplementary Fig. 1

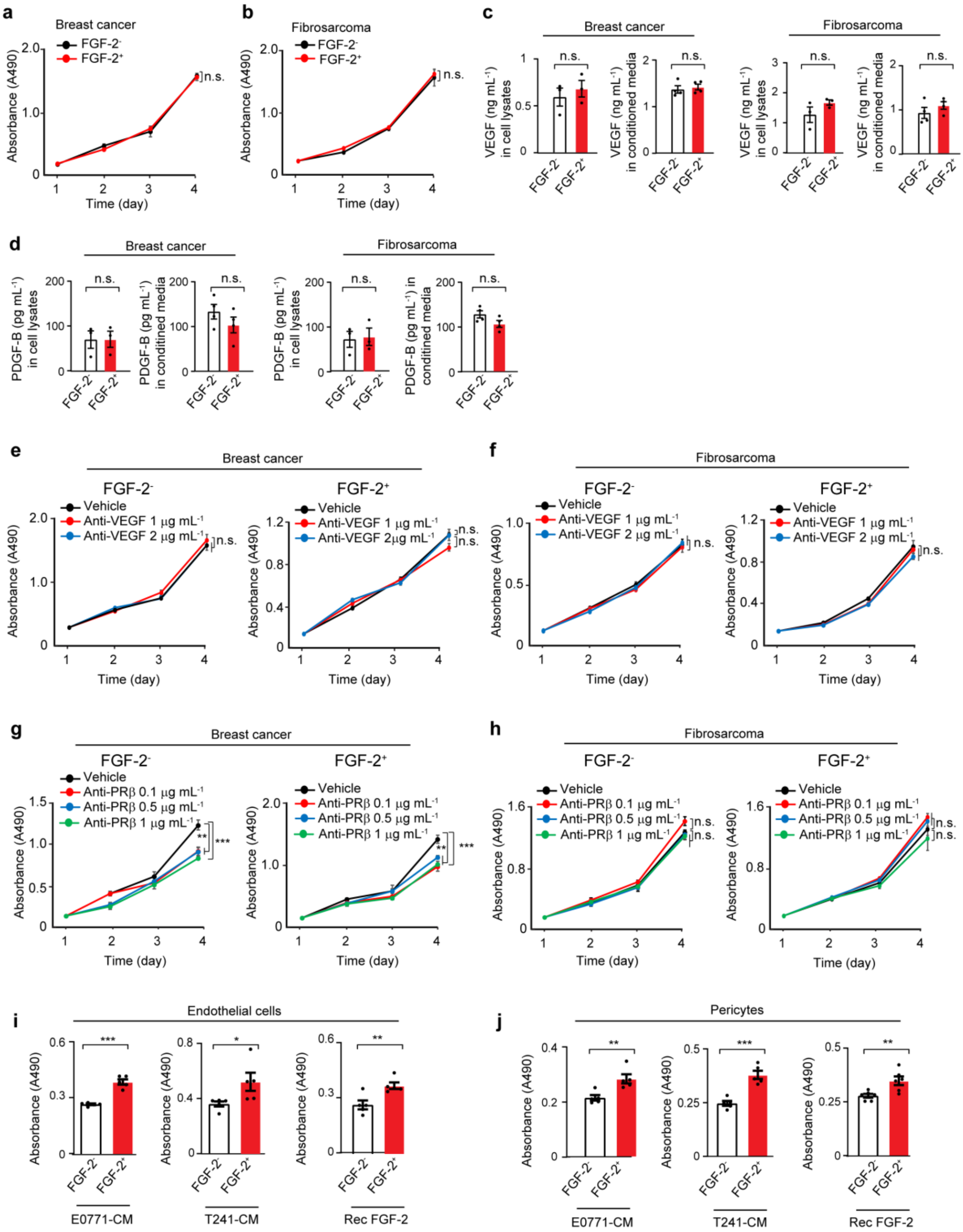

Supplementary Figure 1. Cell proliferation and growth factor production in vitro a. In vitro proliferation of E0771-vector and E0771-FGF-2 cancer cells (n = 6). **b**. In vitro proliferation of T241-vector and T241-FGF-2 cells (n = 6) **c**. ELISA measurement of VEGF levels in E0771-vector and E0771-FGF-2 cancer cell lysates (n = 3 individual samples) and their corresponding conditioned media (n = 4 individual samples), and in T241-vector and T241-FGF-2 cell lysates (n = 3 individual samples) and conditioned media (n = 4 individual samples). **d.** ELISA measurement of PDGF-B levels in E0771-vector and E0771-FGF-2 cell lysates (n = 3)

and conditioned media (n = 4), and in T241-vector and T241-FGF-2 cell lysates (n = 3) and conditioned media (n = 4). **e.** In vitro proliferation of E0771-vector and E0771-FGF-2 cancer cells treated with VEGF blockade (1 and 2 μg mL^{-1}; n(Vector, at Day6) = 6; 6; n(FGF-2, , at Day6) = 5; 6). **f**. In vitro proliferation of T241-vector and T241-FGF-2 cells treated with VEGF blockade (1 and 2 μg mL^{-1}; n = 6). **g**. In vitro proliferation of E0771-vector and E0771-FGF-2 cancer cells treated with PDGFRβ blockade (0.1, 0.5, and 1 μg mL^{-1}; n = 6; *P* = 0.0019, 0.0036, 0.0004) **h**. In vitro proliferation of T241-vector and T241-FGF-2 treated with PDGFRβ blockade (0.1, 0.5, and 1 μg mL^{-1}; n = 6; *P* = 0.0014, 0.0016, 0.0003). **i**. In vitro proliferation of mouse endothelial cells treated with conditioned media from E0771, T241, and recombinant FGF-2 protein (5 ng mL^{-1}) (n = 5; *P* < 0.0001, *P* = 0.046, P = 0.0085). **j**. In vitro proliferation of mouse pericytes treated with conditioned media from E0771, T241 (n = 5), and recombinant FGF-2 protein (50 ng mL^{-1}) (n = 6; *P* = 0.0075, *P* = 0.0003, P = 0.0095). FGF-2^{-} = vector tumor cells; FGF-2^{+} = FGF-2 tumor cells; CM = conditioned media; Rec FGF-2=recombinant FGF-2; n.s. = Not significant; **P* < 0.05; ** *P* < 0.01; *** *P* < 0.001; two-tailed *t*-test. Data presented as mean ± s.e.m. Experiments were repeated two-three times. Source data are provided as a Source Data file.

Supplementary Fig. 2

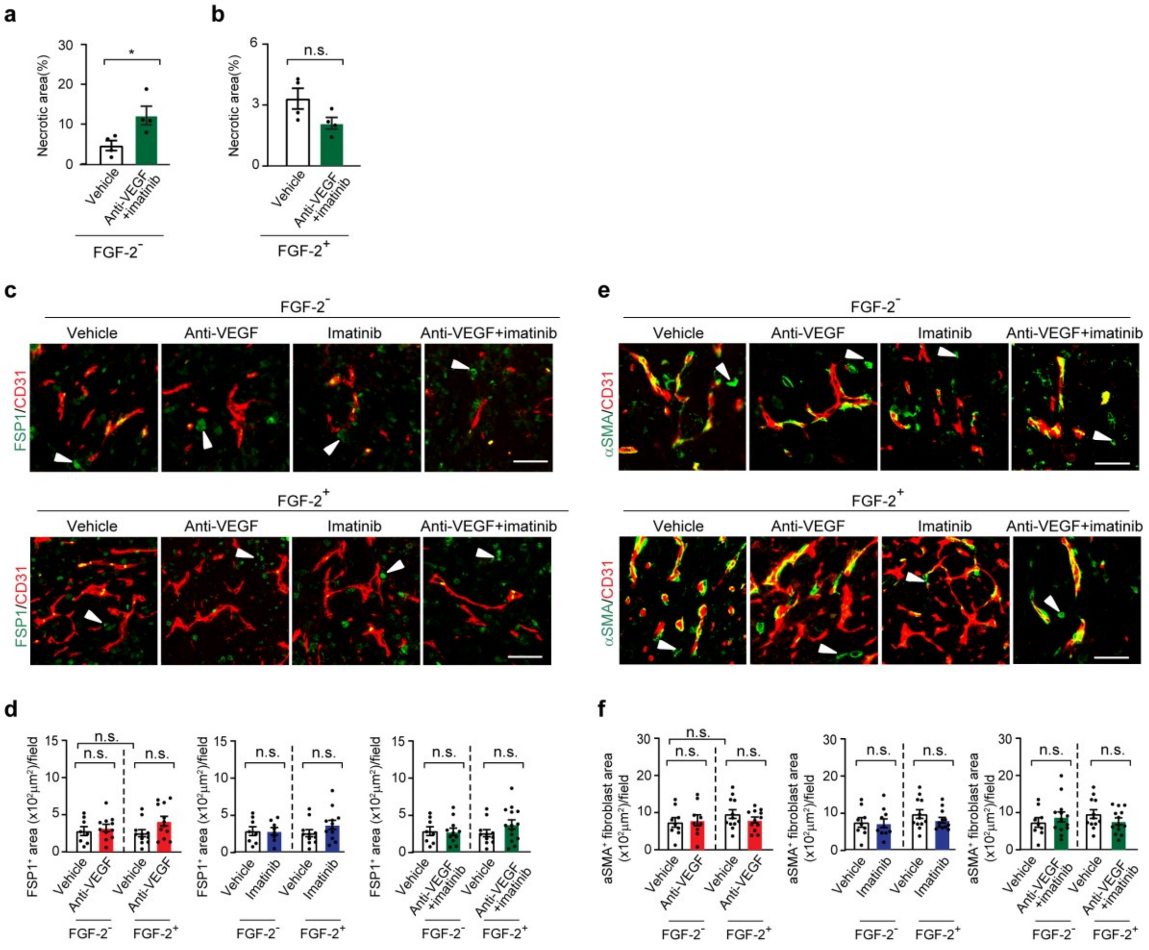

Supplementary Figure 2. Necrosis and cancer-associated fibroblasts in E0771 tumors a. Quantification of the total necrotic areas in vehicle- and anti-VEGF + imatinib dual-treated E0771 breast cancers (n = 4 samples per group; *P*(Vehicle-treated vector vs imatinib plus anti-VEGF-treated vector) = 0.0290). **b.** Quantification of the total necrotic areas in vehicle- and anti-VEGF + imatinib dual-treated E0771-FGF-2 breast cancers (n = 4 samples per group). **c.** CD31^{+} microvessels (red) and fibroblast specific protein-1 (FSP-1)$^{+}$ stromal fibroblasts (green) of anti-VEGF-, imatinib-, and anti-VEGF plus imatinib-treated E0771-vector and E0771-FGF-2 breast cancers. Bar = 50 µm. Arrowheads indicate fibroblasts. **d.** Quantification of FSP-1^{+} fibrotic signals of anti-VEGF-, imatinib- and anti-VEGF plus imatinib-treated E0771-vector and E0771-FGF-2 breast cancers (n(Vector) = 8/11/8/11; n(FGF-2) = 11/12/12/14). **e.** CD31^{+} microvessels (red) and α-smooth muscle actin (α-SMA)$^{+}$ smooth muscle cells and myofibroblasts (green) of anti-VEGF-, imatinib- and anti-VEGF plus imatinib-treated E0771-vector and E0771-FGF-2 breast cancers. Bar = 50 µm. Arrowheads point fibroblasts. **f.** Quantification of α-SMA^{+} myofibrotic signals of anti-VEGF-, imatinib-, and anti-VEGF plus imatinib-treated E0771-vector and E0771-FGF-2 breast cancers (n(Vector) = 9/9/10/13; n(FGF-2) = 12/12/12/13). FGF-2^{-} = vector cancers; FGF-2^{+} = FGF-2 cancers; n.s. = Not significant; *$P < 0.05$; two-tailed *t*-test. Data presented as mean ± s.e.m. Experiments were repeated two times. Source data are provided as a Source Data file.

Supplementary Fig. 3

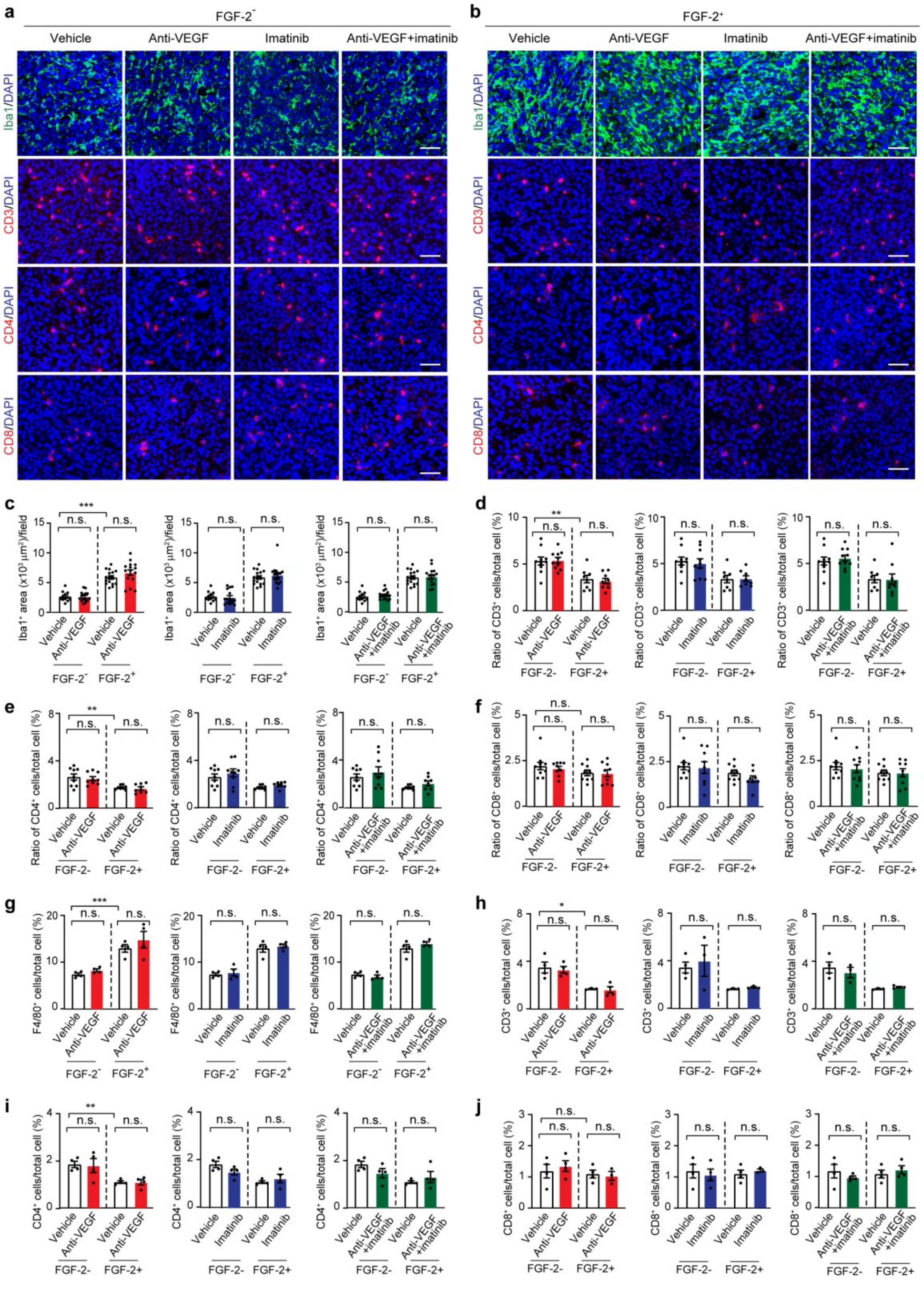

Supplementary Figure 3. Tumor inflammatory cells and immune cells in E0771 breast cancers. a, b. Immunohistochemical analysis of Iba 1^{+} inflammatory macrophages (green), $CD3^{+}$ total T cell population (red), $CD4^{+}$ subpopulation of T cells (red), and $CD8^{+}$ subpopulation of T cells (red) in anti-VEGF-, imatinib- and anti-VEGF plus imatinib-treated E0771-vector and E0771-FGF-2 breast cancers. Bar = 50 μm. **c.** Quantification of Iba 1^{+} inflammatory macrophages of anti-VEGF-,

imatinib- and anti-VEGF plus imatinib-treated E0771-vector and E0771-FGF-2 breast cancers (n(Vector) = 12/15/15/16; n(FGF-2) = 16 each; *P*(Vector vs FGF-2) < 0.0001). **d** Quantification of $CD3^+$ T cells of anti-VEGF-, imatinib- and anti-VEGF plus imatinib-treated E0771-vector and E0771-FGF-2 breast cancers (n(Vector) = 10 each; n(FGF-2) = 8/9/8/9; *P*(Vector vs FGF-2) = 0.0052). **e** Quantification of $CD4^+$ T cells of anti-VEGF-, imatinib- and anti-VEGF plus imatinib-treated E0771-vector and E0771-FGF-2 breast cancers (n(Vector) = 9 each; n(FGF-2) = 8 each; *P*(Vector vs FGF-2) = 0.0060). **f** Quantification of $CD8^+$ T cells of anti-VEGF-, imatinib- and anti-VEGF plus imatinib-treated E0771-vector and E0771-FGF-2 breast cancers (n(Vector) = 10/9/9/10; n(FGF-2) = 9/9/8/9 each). **g-j.** FACS measurement of $F4/80^+$ inflammatory macrophages (n = 4 each; *P*(Vector vs FGF-2) = 0.0007) (**g**), $CD3^+$ T cells (n(Vector) = 4/4/3/3; n(FGF-2) = 3/4/3/4; *P*(Vector vs FGF-2) = 0.0211) (**h**), $CD4^+$ T cells (n = 4 each; *P*(Vector vs FGF-2) = 0.002) (**i**), and $CD8^+$ T cells (n(Vector) = 4 each; n(FGF-2) = 4/3/3/4). (**j**) of anti-VEGF-, imatinib- and anti-VEGF plus imatinib-treated E0771-vector and E0771-FGF-2 breast cancers. The values are presented as the percentage of positive signals versus the total gated events. $FGF\text{-}2^-$ = vector cancers; $FGF\text{-}2^+$ = FGF-2 cancers; n.s. = Not significant; *$P < 0.05$, **$P < 0.01$, ***$P < 0.001$; two-tailed *t*-test. Data presented as mean ± s.e.m. Experiments were repeated two times. Source data are provided as a Source Data file.

Supplementary Fig. 4

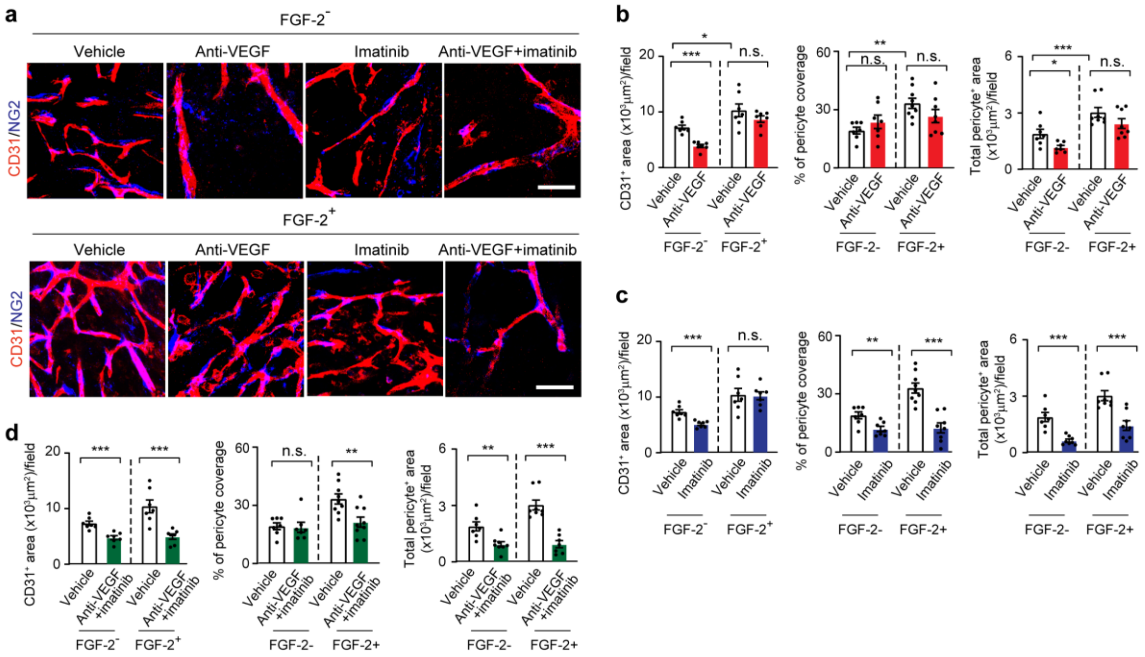

Supplementary Figure 4. Tumor angiogenesis and perivascular coverage of anti-VEGF-, imatinib-, and anti-VEGF plus imatinib-treated fibrosarcomas.

a. CD31$^+$ (red) and NG2$^+$ (blue) microvessels in anti-VEGF-, imatinib- and anti-VEGF plus imatinib-treated fibrosarcomas. Bar = 100 μm. **b.** Quantification of microvessels (n = 7 each; *P*(Vector vs FGF-2) = 0.0275; *P*(Vehicle-treated vector vs anti-VEGF-treated vector) < 0.001), pericyte coverages (n = 7/7/8/8; *P*(Vector vs anti-FGF-2) = 0.0016) and pericyte area (n = 7/7/8/8; *P*(Vector vs anti-FGF-2) = 0.0009; *P*(Vehicle-treated vector vs anti-VEGF-treated vector) = 0.0313) of vehicle- anti-VEGF-treated fibrosarcomas. **c.** Quantification of microvessels (n = 7 each; *P*(Vehicle-treated vector vs imatinib-treated vector) = 0.0004), pericyte coverages (n = 7/7/8/8; *P*(Vehicle-treated vector vs imatinib-treated vector) = 0.0084; *P*(Vehicle-treated FGF-2 vs imatinib-treated FGF-2) = 0.0001) and pericyte area (n = 7/7/8/8; *P*(Vehicle-treated vector vs imatinib-treated vector) < 0.0001; *P*(Vehicle-treated FGF-2 vs imatinib-treated FGF-2) = 0.0008) of vehicle- and imatinib-treated fibrosarcomas. **d.** Quantification of microvessels (n = 7 each; *P*(Vehicle-treated vector vs combination-treated vector) = 0.0004; *P*(Vehicle-treated FGF-2 vs combination-treated FGF-2) = 0.00096), pericyte coverages (n = 7/7/8/8; *P*(Vehicle-treated FGF-2 vs combination-treated FGF-2) = 0.0031) and pericyte area (n = 7/8/8/7; *P*(Vehicle-treated vector vs combination-treated vector) = 0.0059; *P*(Vehicle-treated FGF-2 vs combination-treated FGF-2) < 0.0001) of vehicle- and anti-VEGF plus imatinib-treated fibrosarcomas. FGF-2$^-$ = vector cancers; FGF-2$^+$ = FGF-2 cancers; n.s. = Not significant; *$P < 0.05$; **$P < 0.01$; ***$P < 0.001$; two-tailed *t*-test. Data presented as mean ± s.e.m. Experiments were repeated two times. Source data are provided as a Source Data file.

Supplementary Fig. 5

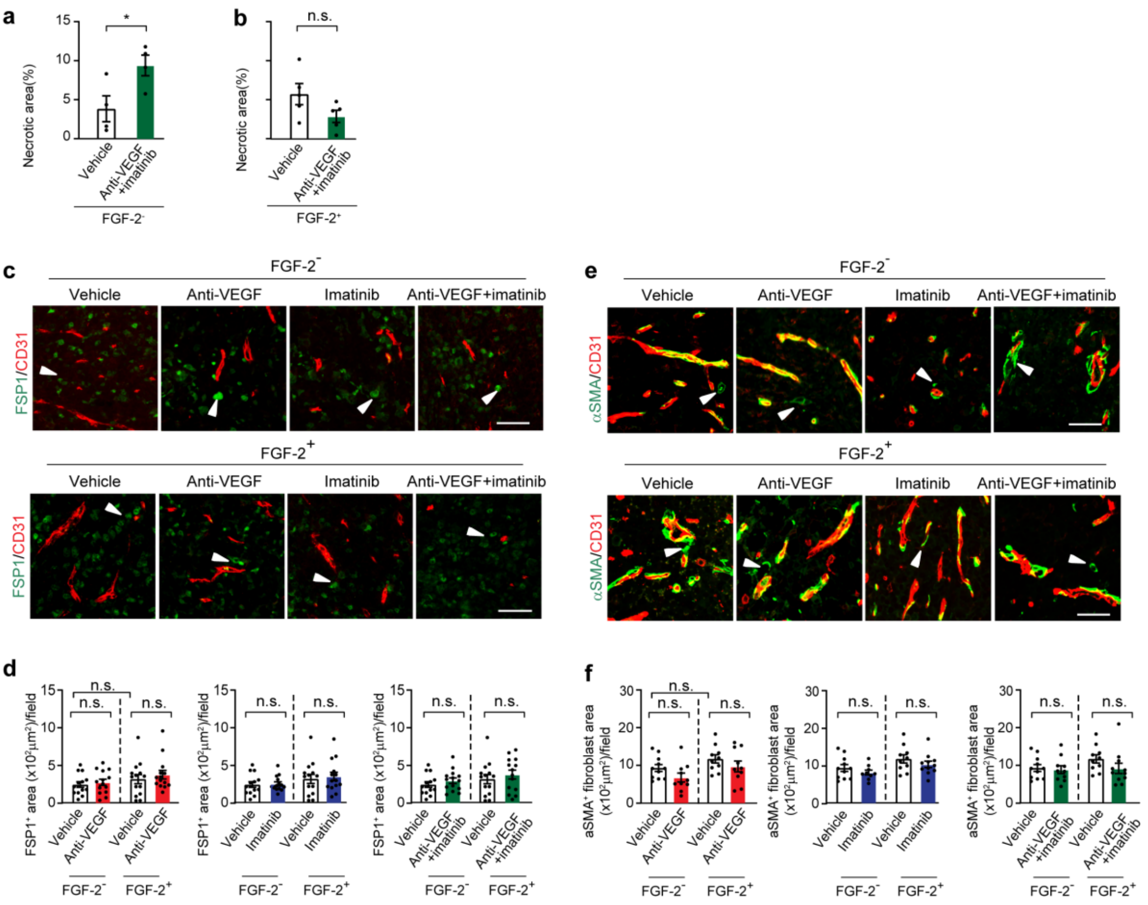

Supplementary Figure 5. Necrosis and cancer-associated fibroblasts in fibrosarcomas a. Quantification of the total necrotic areas in vehicle- and anti-VEGF + imatinib dual-treated T241-fibrosarcomas (n = 4 samples per group; *P*(Vehicle-treated vector vs imatinib plus anti-VEGF-treated vector) = 0.0398). **b.** Quantification of the total necrotic areas in vehicle- and anti-VEGF + imatinib dual-treated T241-FGF-2 fibrosarcomas (n = 5 samples per group). **c.** CD31$^+$ microvessels (red) and fibroblast specific protein-1 (FSP-1)$^+$ stromal fibroblasts (green). Arrowheads indicate fibroblasts. Bar = 50 μm. **d.** Quantification of FSP-1$^+$ fibrotic signals of anti-VEGF-, imatinib- and anti-VEGF plus imatinib-treated T241-vector and T241-FGF-2 fibrosarcomas (n = 14 samples per group). **e.** CD31$^+$ microvessels (red) and α-smooth muscle actin (α-SMA)$^+$ smooth muscle cells and myofibroblasts (green) in anti-VEGF-, imatinib- and anti-VEGF plus imatinib-treated T241-vector and T241-FGF-2 fibrosarcomas. Arrowheads point fibroblasts. Bar = 50 μm. **f.** Quantification of α-SMA$^+$ myofibrotic signals of anti-VEGF-, imatinib- and anti-VEGF plus imatinib-treated T241-vector and T241-FGF-2 fibrosarcomas (n(Vector) = 11/10/9/10; n(FGF-2) = 10/10/11/11). FGF-2$^-$ = vector cancers; FGF-2$^+$ = FGF-2 cancers; n.s. = Not significant; *$P < 0.05$; two-tailed *t*-test. Data presented as mean ± s.e.m. Experiments were repeated two times. Source data are provided as a Source Data file.

Supplementary Fig. 6

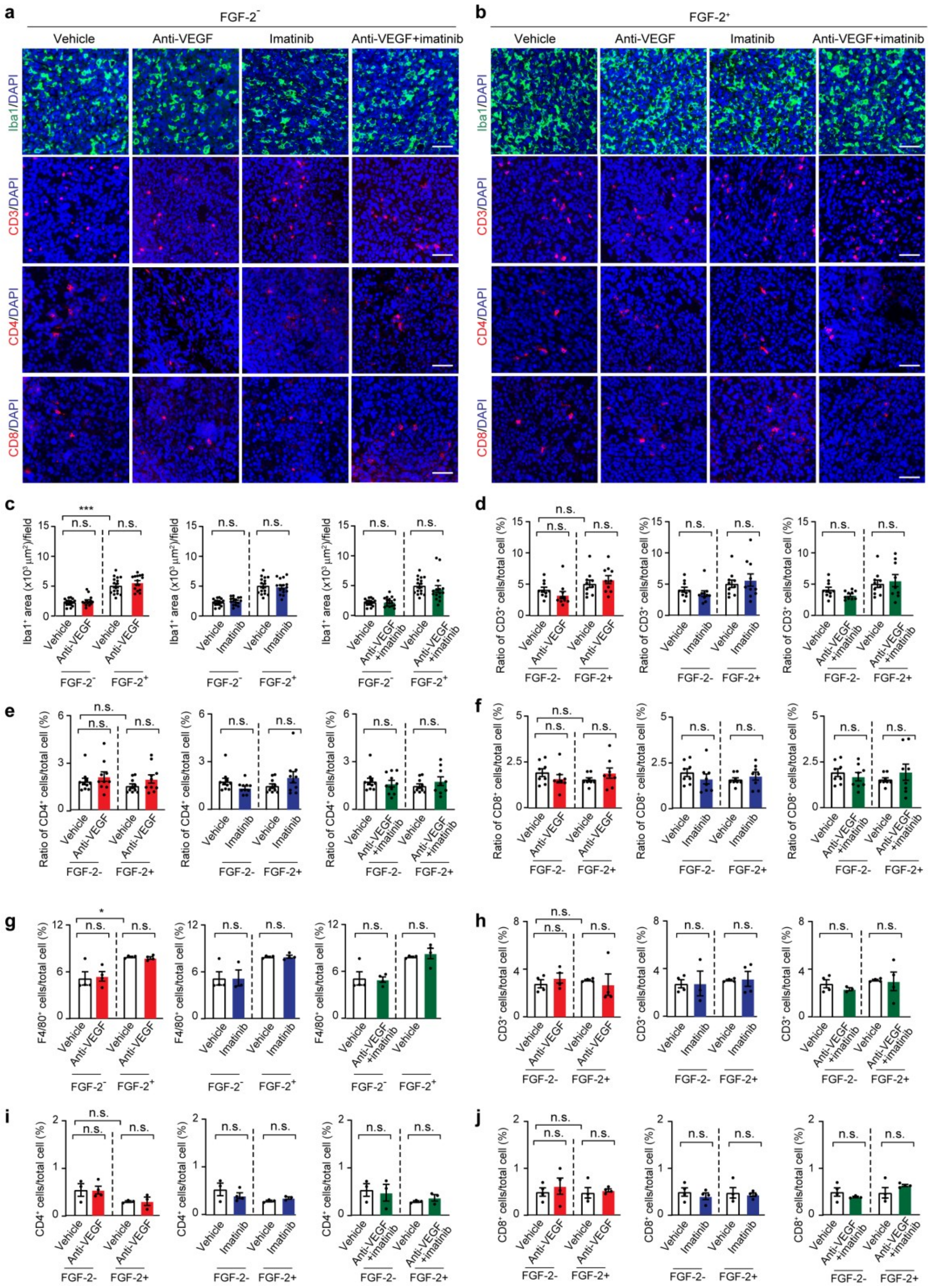

Supplementary Figure 6. Tumor inflammatory cells and immune cells in T241 fibrosarcomas. a, b. Immunohistochemical analysis of Iba 1^+ inflammatory macrophages (green), $CD3^+$ total T cell population (red), $CD4^+$ subpopulation of T cells (red), and $CD8^+$ subpopulation of T cells (red) in anti-VEGF-, imatinib- and anti-VEGF plus imatinib-treated T241-vector and T241-FGF-2 fibrosarcomas. Bar = 50 µm. **c.** Quantification of Iba 1^+ inflammatory macrophages of anti-VEGF-,

imatinib- and anti-VEGF plus imatinib-treated T241-vector and T241-FGF-2 fibrosarcomas (n(Vector) = 16/14/16/16; n(FGF-2) = 16/15/16/15; *P*(Vector vs FGF-2) < 0.0001). **d** Quantification of $CD3^+$ T cells of anti-VEGF-, imatinib-, and anti-VEGF plus imatinib-treated T241-vector and T241-FGF-2 fibrosarcomas (n(Vector) = 10 each; n(FGF-2) = 10/10/10/9). **e** Quantification of $CD4^+$ T cells of anti-VEGF-, imatinib-, and anti-VEGF plus imatinib-treated T241-vector and T241-FGF-2 fibrosarcomas (n(Vector) = 10 each; n(FGF-2) = 10/10/10/9). **f** Quantification of $CD8^+$ T cells of anti-VEGF-, imatinib-, and anti-VEGF plus imatinib-treated T241-vector and T241-FGF-2 fibrosarcomas (n = 8 samples per group). **g-j.** FACS measurement of $F4/80^+$ inflammatory macrophages (n(Vector) = 4/4/3/4; n(FGF-2) = 4 each; *P*(Vector vs FGF-2) = 0.0176) (**g**), $CD3^+$ T cells (n(Vector) = 4/4/3/3; n(FGF-2) = 4 each) (**h**), $CD4^+$ T cells (n(Vector) = 3/4/4/3; n(FGF-2) = 3 each) (**i**), and $CD8^+$ T cells(n(Vector) = 4/4/4/3; n(FGF-2) = 4 each) (**j**) of anti-VEGF-, imatinib- and anti-VEGF plus imatinib-treated T241-vector and T241-FGF-2 fibrosarcomas. The values are presented as the percentage of positive signals versus the total gated events. $FGF\text{-}2^-$ = vector cancers; $FGF\text{-}2^+$ = FGF-2 cancers; n.s. = Not significant; **P* < 0.05, ****P* < 0.001; two-tailed *t*-test. Data presented as mean ± s.e.m. Experiments were repeated two times. Source data are provided as a Source Data file.

Supplementary Fig. 7

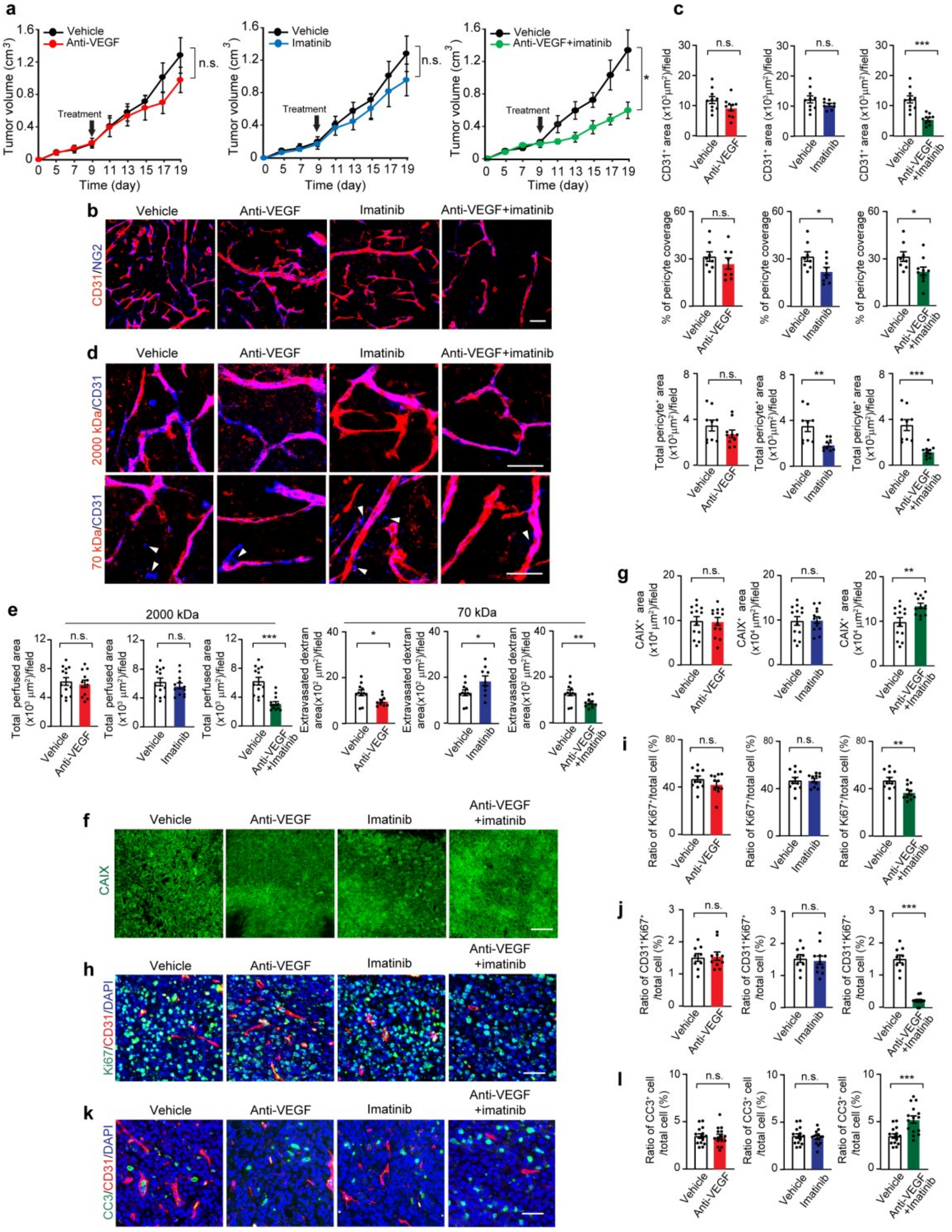

Supplementary Figure 7. Tumor growth, vascular function, and hypoxia in various drug-treated established FGF-2^{+} fibrosarcomas. a. Drug treatment was initiated at day 9 when tumors took off. Tumor growth of vehicle-, anti-VEGF-, imatinib- and anti-VEGF plus imatinib- treated T241-FGF-2 fibrosarcomas (n = 7/5/6/5; *P*(Vehicle vs imatinib plus anti-VEGF) = 0.0245). **b.** CD31^{+} microvessels (red) and NG2^{+} pericytes (blue) in various drug-treated T241-FGF-2 fibrosarcomas. Bar = 100 µm. **c.** Quantification of microvessels (n = 10/10/9/10; *P*(Vehicle vs imatinib plus anti-VEGF) < 0.0001), pericyte coverages (n = 8/8/9/10; Vehicle vs imatinib) = 0.0290; Vehicle vs imatinib plus anti-VEGF) = 0.0336) and pericyte area

(n = 9/10/9/10; Vehicle vs imatinib) = 0.0052; *P*(Vehicle vs imatinib plus anti-VEGF) = 0.0001) of vehicle-, anti-VEGF-, imatinib- and anti-VEGF plus imatinib- treated T241-FGF-2. **d.** Vascular perfusion of 2000 kDa dextran (red) and vascular permeability of 70 kDa dextran (red) of various therapy-treated T241-FGF-2. Bar = 50 μm. **e.** Quantification of vascular perfusion and permeability of vehicle-, anti-VEGF-, imatinib- and anti-VEGF plus imatinib- treated T241-FGF-2 (Perfusion: n = 13/12/12/13; *P*(Vehicle vs imatinib plus anti-VEGF) < 0.0001; permeability: n = 11/9/9/11; *P*(Vehicle vs anti-VEGF) = 0.0342 ; *P*(Vehicle vs imatinib) = 0.0373 ; *P*(Vehicle vs imatinib plus anti-VEGF) = 0.0067). **f.** $CAXI^+$ signals (green) of tumor hypoxia. Bar = 100 μm. **g.** Quantification of $CAXI^+$ hypoxic signals of various therapy-treated T241-FGF-2 (n = 13 each; *P*(Vehicle vs imatinib plus anti-VEGF) = 0.0036). **h.** $Ki67^+$ proliferative cell signals (green) stained with $CD31^+$ microvessels (red) and DAPI (blue) of various therapy-treated T241-FGF-2. **i.** Quantification of $Ki67^+$ signals in various therapy-treated T241-FGF-2 (n = 11 each; *P*(Vehicle vs imatinib plus anti-VEGF) = 0.0042). **j.** Quantification of $Ki67^+$ and $CD31^+$ double positive signals in various therapy-treated T241-FGF-2 (n = 9/10/11/11; *P*(Vehicle vs imatinib plus anti-VEGF) < 0.0001). **k.** Micrographs of caspase-3^+ apoptotic cells (green) in various therapy-treated T241-FGF-2. Bar = 50 μm. **l.** Quantification of caspase-3 signals in various therapy-treated T241-FGF-2 fibrosarcomas (n = 15 each; *P*(Vehicle vs imatinib plus anti-VEGF) = 0.0005). n.s. = Not significant; $*P < 0.05$; $**P < 0.01$; $***P < 0.001$; two-tailed *t*-test. Data presented as mean ± s.e.m.

Supplementary Fig. 8

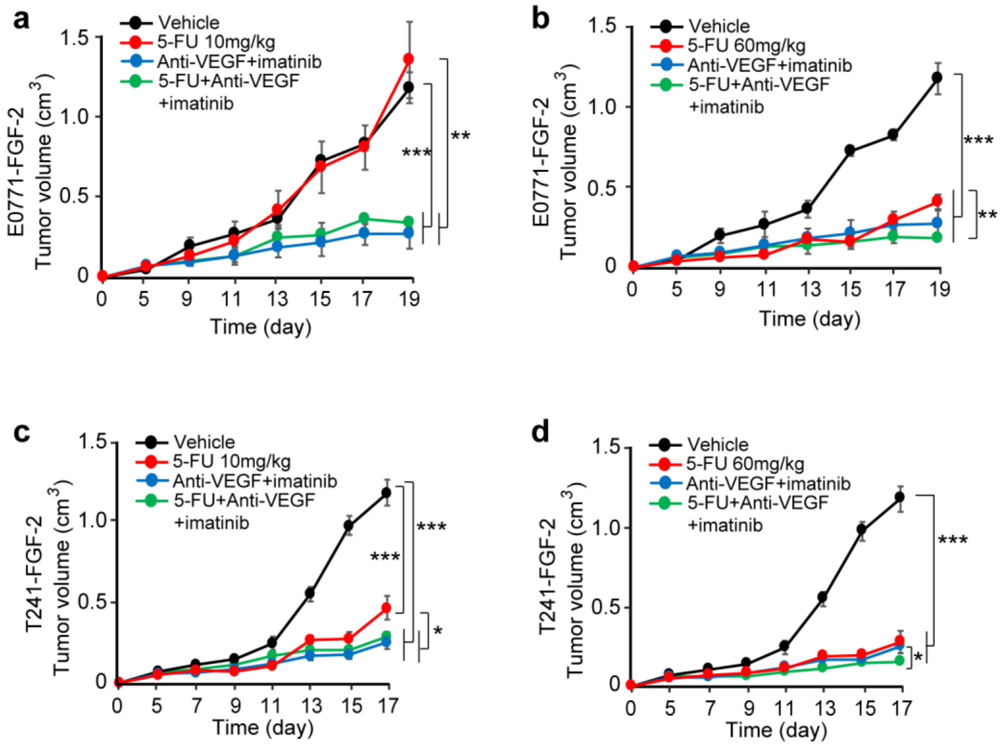

Supplementary Figure 8. Triple Combination therapy with chemotherapeutics. a. Tumor growth of vehicle-, anti-VEGF plus imatinib-, 5-FU (10 mg kg-1)-, and the triple combination of 5-FU and anti-VEGF plus imatinib-treated E0771-FGF-2 breast cancers (n = 4 animals per group; *P*(Vehicle vs imatinib plus anti-VEGF) = 0.0005; *P*(Vehicle vs the triple combination of 5-FU and anti-VEGF plus imatinib) = 0.0002; *P*(5FU vs imatinib plus anti-VEGF) = 0.0054; *P*(5-FU vs the triple combination of 5-FU and anti-VEGF plus imatinib = 0.0056). **b.** Tumor growth of vehicle-, anti-VEG plus imatinib-, 5-FU (60 mg kg-1)-, and the combination of 5-FU and anti-VEGF plus imatinib-treated E0771-FGF-2 breast cancers (n = 4 animals per group; *P*(Vehicle vs imatinib plus anti-VEGF) = 0.0005; *P*(Vehicle vs the triple combination of 5-FU and anti-VEGF plus imatinib) < 0.0001; *P*(Vehicle vs 5-FU) = 0.0003; *P*(5-FU vs the triple combination of 5-FU and anti-VEGF plus imatinib) = 0.0024). **c.** Tumor growth of vehicle-, anti-VEGF plus imatinib-, 5-FU (10 mg kg-1)-, and the combination of 5-FU and anti-VEGF plus imatinib-treated T241-FGF-2 fibrosarcomas (n = 4 animals in vehicle- and anti-VEGF plus imatinib- treated groups. n = 5 animals in 5-FU and the combination of 5-FU and anti-VEGF plus imatinib-treated groups. two-site injections per animal; *P*(Vehicle vs imatinib plus anti-VEGF) < 0.0001; *P*(Vehicle vs 5-FU) < 0.0001; *P*(Vehicle vs the triple combination of 5-FU and anti-VEGF plus imatinib) < 0.0001; *P*(5-FU vs anti-VEGF plus imatinib) = 0.0211; *P*(5-FU vs the triple combination of 5-FU and anti-VEGF plus imatinib) = 0.0195). **d.** Tumor growth of vehicle-, anti-VEGF plus imatinib-, 5-FU (60 mg kg-1)-, and the combination of 5-FU and anti-VEGF plus imatinib-treated T241-FGF-2 fibrosarcomas. (n = 4 animals in vehicle- and anti-VEGF plus imatinib-treated groups. n = 5 animals in 5-FU-, and the

combination of 5-FU and anti-VEGF plus imatinib-treated, two-site injections per animal; *P*(Vehicle vs imatinib plus anti-VEGF) < 0.0001; *P*(Vehicle vs 5-FU) < 0.0001; *P*(Vehicle vs the triple combination of 5-FU and anti-VEGF plus imatinib) < 0.0001; *P*(Imatinib plus anti-VEGF vs the triple combination of 5-FU and anti-VEGF plus imatinib) = 0.0403); two-tailed *t*-test. Data presented as mean ± s.e.m.

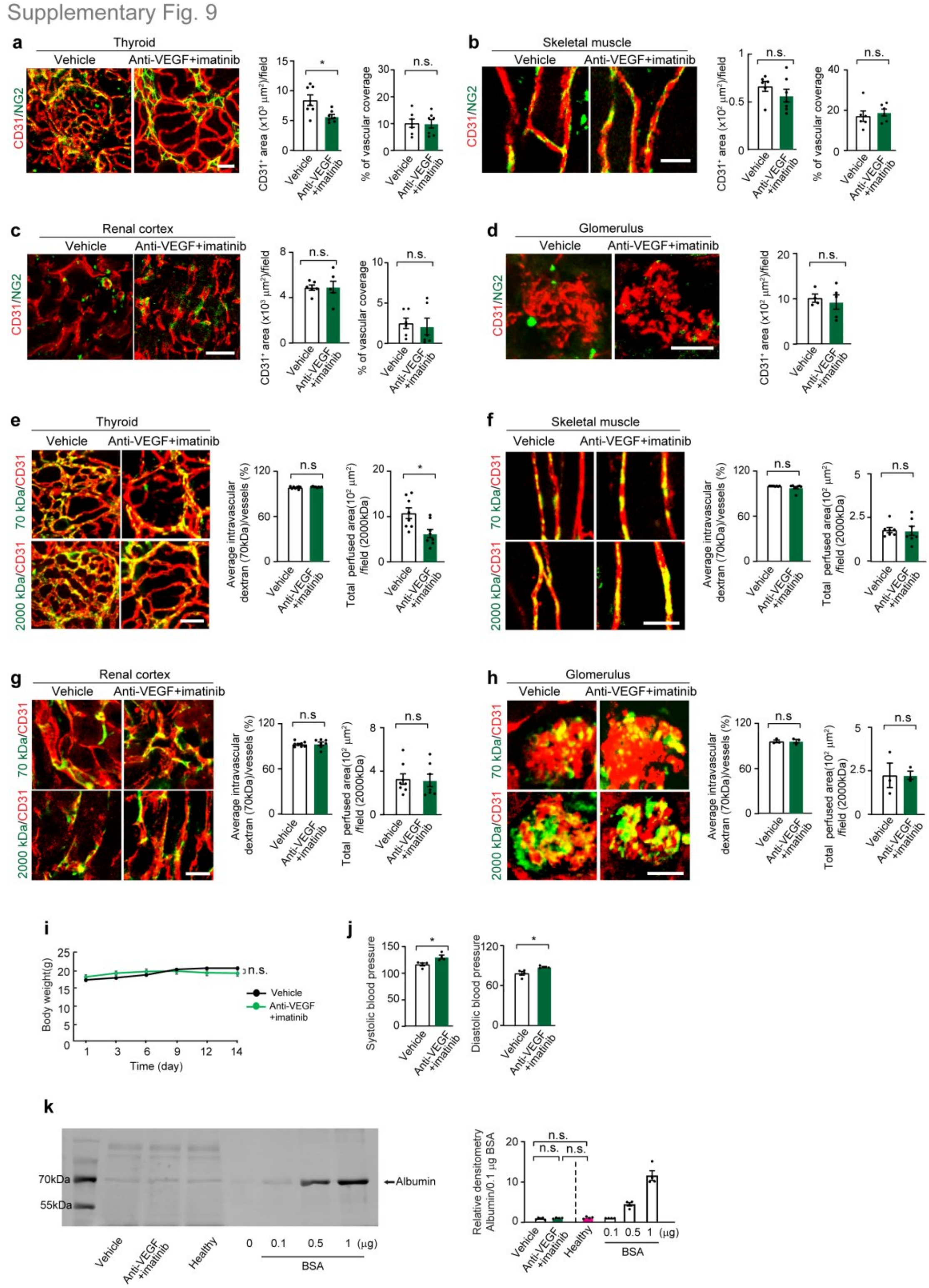

Supplementary Figure 9. Systemic effects of anti-VEGF plus imatinib therapy on healthy vasculatures. (**a-d**) Immunohistochemical images of CD31^{+} microvessels (red) and NG2^{+} pericytes (green) and quantification of microvessel density and pericyte coverage of vehicle- and anti-VEGF plus imatinib-treated healthy mice. **a**. Treatment impact on thyroid tissues (n = 7 samples per groups; *P*(CD31^{+} microvessels: Vehicle-treated vs imatinib plus anti-VEGF-treated) = 0.0153). **b**. Treatment impact on skeletal muscle tissues (n = 6 samples per groups). **c**. Treatment

impact on renal cortex (n = 6 samples per groups). **d**. Treatment impact on glomeruli (n = 4 in vehicle, n = 5 in combination-treatment group). (**e-h**) Vascular permeability of fluorescein-labeled lysinated 70 kDa dextran (green) and vascular perfusion of fluorescein-labeled lysinated 2000 kDa dextran (green) stained with $CD31^+$ microvessels (red). Quantification of vascular permeability and perfusion of vehicle- and anti-VEGF plus imatinib-treated groups in various organs. **e**. Treatment impact on thyroid tissues (n = 8 samples per groups; *P*(Vascular permeability: Vehicle-treated vs combination) = 0.0102). **f**. Treatment impact on skeletal muscle tissues (n = 8 in permeability, n = 7 in perfusion). **g**. Treatment impact on renal cortex (n = 8 in vehicle, n = 7 in combination-treatment). **h**. Treatment impact on glomeruli (n = 3 samples each). **i.** Total body weight of C57BL/6 of vehicle- and anti-VEGF plus imatinib-treated groups (n = 5 animals in vehicle, n = 4 animals in combination-treatment group). **j.** Systolic and diastolic blood pressure changes after 2-week treatment with vehicle, and anti-VEGF plus imatinib (n = 4 animals per group; *P*(Systolic: Vehicle-treated vs imatinib plus anti-VEGF-treated) = 0.0212; *P*(Diastolic: Vehicle-treated vs imatinib plus anti-VEGF-treated) = 0.02150). **k.** SDS-PAGE gel electrophoresis of urine samples. BSA was used as a marker for albumin. Quantification of the urine proteins (n = 5 animals in vehicle, n = 4 animals in combination-treatment group). Urine from non-treated healthy mice (n = 4 animals) was used as a control. All scale bar = 25 μm. n.s. = Not significant; *$P < 0.05$; two-tailed *t*-test. Data presented as mean ± s.e.m. Experiments were repeated two times. Source data are provided as a Source Data file.

Supplementary Fig. 10

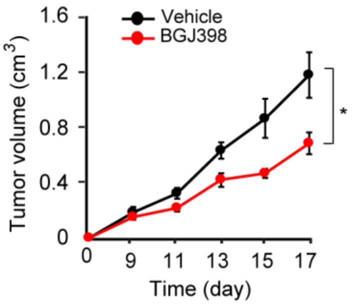

Supplementary Figure 10. Tumor growth of anti-FGFR-treated FGF-2^{+} fibrosarcomas. Tumor growth of vehicle- (n = 4 animals per group; *P* = 0.0223) and BGJ398-treated (n = 6 animals per group) T241-FGF-2 fibrosarcomas. *$P < 0.05$; two-tailed *t*-test. Data presented as mean ± s.e.m. Experiments were repeated two times. Source data are provided as a Source Data file.

Supplementary Fig. 11

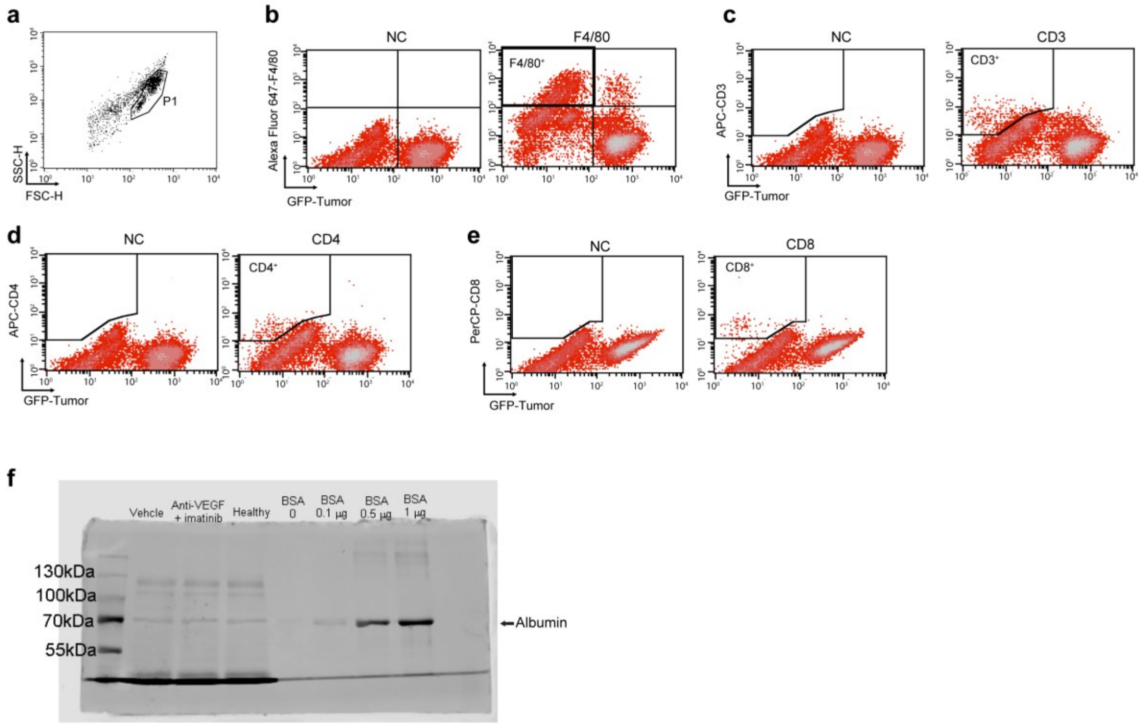

Supplementary Figure 11. Gating strategies for flow cytometry and a full scan image. Gating strategy used for flow cytometry analysis. **a.** FSC-H/SSC-H gate for exclusion of debris. Gates for F4/80$^+$ inflammatory macrophages (**b**), CD3$^+$ T cells (**c**), CD4$^+$ T cells (**d**), and CD8$^+$ T cells (**e**). NC= negative control. **f.** Full scan data of immunoblot used for supplementary figure 9k.
