# Supplementary material for: Therapeutic paradigm of dual targeting VEGF and PDGF for effectively treating FGF-2 off-target tumors
Source: Nat Commun. 2020 Jul 24;11:3704. doi: 10.1038/s41467-020-17525-6 (PMC7382445; doi:10.1038/s41467-020-17525-6)
Supplement: Supplementary file 3 — Reporting Summary [file 41467_2020_17525_MOESM3_ESM.pdf]

nature research

Corresponding author(s): Yihai Cao

Last updated by author(s): Jun 25, 2020

Reporting Summary

Nature Research wishes to improve the reproducibility of the work that we publish. This form provides structure for consistency and transparency in reporting. For further information on Nature Research policies, see Authors & Referees and the Editorial Policy Checklist .

Statistics

For all statistical analyses, confirm that the following items are present in the figure legend, table legend, main text, or Methods section.

n/a	Confirmed	
☐	☒	The exact sample size (*n*) for each experimental group/condition, given as a discrete number and unit of measurement
☐	☒	A statement on whether measurements were taken from distinct samples or whether the same sample was measured repeatedly
☐	☒	The statistical test(s) used AND whether they are one- or two-sided<br>*Only common tests should be described solely by name; describe more complex techniques in the Methods section.*
☒	☐	A description of all covariates tested
☒	☐	A description of any assumptions or corrections, such as tests of normality and adjustment for multiple comparisons
☐	☒	A full description of the statistical parameters including central tendency (e.g. means) or other basic estimates (e.g. regression coefficient) AND variation (e.g. standard deviation) or associated estimates of uncertainty (e.g. confidence intervals)
☐	☒	For null hypothesis testing, the test statistic (e.g. *F*, *t*, *r*) with confidence intervals, effect sizes, degrees of freedom and *P* value noted<br>*Give P values as exact values whenever suitable.*
☒	☐	For Bayesian analysis, information on the choice of priors and Markov chain Monte Carlo settings
☒	☐	For hierarchical and complex designs, identification of the appropriate level for tests and full reporting of outcomes
☒	☐	Estimates of effect sizes (e.g. Cohen's *d*, Pearson's *r*), indicating how they were calculated

Our web collection on statistics for biologists contains articles on many of the points above.

Software and code

Policy information about availability of computer code

Data collection	Imaging data were collected using EZ-C1 3.91 (confocal microscopy), NIS-Elements D3.2 (fluorescence microscopy), NIS-Elements F 3.0 (light microscopy), and gel data were collected by Image Studio Ver3.1. For the collection of Flow Cytometry data, BD Cell Quest Pro version 6.0 was used.
Data analysis	Imaging data were analyzed using Photoshop software CS6 version13.0, ImageJ 1.52a, Microsoft Excel 2016, and Graphpad Prism 8.4.2. Gel data were analyzed using Image Studio Ver3.1 and Microsoft Excel 2016. For Flow Cytometry data analysis, BD Cell Quest Pro version 6.0 was used.

For manuscripts utilizing custom algorithms or software that are central to the research but not yet described in published literature, software must be made available to editors/reviewers. We strongly encourage code deposition in a community repository (e.g. GitHub). See the Nature Research guidelines for submitting code & software for further information.

Data

Policy information about availability of data

All manuscripts must include a data availability statement. This statement should provide the following information, where applicable:

- Accession codes, unique identifiers, or web links for publicly available datasets
- A list of figures that have associated raw data
- A description of any restrictions on data availability

All raw data were provided as a Source Data File.
Data supporting the findings of this study are available within the article and its Supplementary Information files. The source data underling Figs. 1a-e, g-i, 2c, d, f, 3b, c, e, f, 4a-d, g, h, j, 5b, c, e, f, 6a, c, e, g, i, j, l, m, and Supplementary Figs. 1, 2a, b, d, f, 3c-j, 4b-d, 5a, b, d, f, 6c-j, 7a, c, e, g, i, j, l, 8, 9, and 10 are provided as a Source Data file. The Cancer Genome Atlas data sets for the survival study are downloaded from the link;
http://gdac.broadinstitute.org/runs/
The values used for the survival study are provided as a Source Data file (Fig 7a-d).

Field-specific reporting

Please select the one below that is the best fit for your research. If you are not sure, read the appropriate sections before making your selection.

- [x] Life sciences
- [ ] Behavioural & social sciences
- [ ] Ecological, evolutionary & environmental sciences

For a reference copy of the document with all sections, see nature.com/documents/nr-reporting-summary-flat.pdf

Life sciences study design

All studies must disclose on these points even when the disclosure is negative.

Sample size	Sample numbers in vivo and in vitro experiments were indicated according to our published data<br>Proc Natl Acad Sci U S A. 2017 Jun 27;114(26):E5226-E5235. doi: 10.1073/pnas.1<br>Proc Natl Acad Sci U S A. 2017 Nov 7;114(45):E9635-E9644. doi: 10.1073/pnas.1703431114. Epub 2017 Oct 23.<br>Cell Metab. 2018 Jul 3;28(1):104-117.e5. doi: 10.1016/j.cmet.2018.05.005. Epub 2018 May 31.<br>Due to the 3R principle for our recent animal ethical permission, we used a minimal number of animals, which was still possible for general statistics.
Data exclusions	When the animal condition does not match the approved animal ethical, animals were not participated in the data collection. The criteria of animal health was pre-established following the approved animal ethical.
Replication	All experiments except drug treatments with a chemotherapeutic drug were repeated. In vitro data were repeated 2-3 times. All attempts at replication were successful.<br>The combination of anti-angiogenic therapy and chemotherapeutic drugs was well studied in our and others' works.<br>Ma J. Et al Mol Cancer Ther. 2008; 7(12): 3670–3684.<br>Zhang Y. et al Proc Natl Acad Sci U S A. 2017;114(26)<br>Hurwitz H. et al. N Engl J Med. 2004;350(23):2335-42.<br>In addition to these works, the combination effect with 5-FU and anti-angiogenic therapy was confirmed in our settings.
Randomization	Age or gender-matched mice were randomly allocated to the groups. In the cell-based assay, groups were divided randomly.
Blinding	The researchers were blinded during the data collection and data analysis of in vivo and in vitro experiments, apart from the animal treatment time. Due to the different administration methods of drug treatment, it was not possible to be blinded completely during animal treatment.

Reporting for specific materials, systems and methods

We require information from authors about some types of materials, experimental systems and methods used in many studies. Here, indicate whether each material, system or method listed is relevant to your study. If you are not sure if a list item applies to your research, read the appropriate section before selecting a response.

Materials & experimental systems

n/a	Involved in the study	
[ ]	[x]	Antibodies
[ ]	[x]	Eukaryotic cell lines
[x]	[ ]	Palaeontology
[ ]	[x]	Animals and other organisms
[x]	[ ]	Human research participants
[x]	[ ]	Clinical data

Methods

n/a	Involved in the study	
[x]	[ ]	ChIP-seq
[ ]	[x]	Flow cytometry
[x]	[ ]	MRI-based neuroimaging

Antibodies

Antibodies used	IHC<br>Anti-CD31 (1:200; 553370; BD Pharmingen), https://www.bdbiosciences.com/eu/applications/research/stem-cell-research/cancer-research/mouse/purified-rat-anti-mouse-cd31-mec-133/p/553370<br>Anti-NG2 (1:200; AB5320; Merck), https://www.sigmaaldrich.com/catalog/product/mm/ab5320?lang=en&region=SE&gclid=CjwKCAjw88v3BRBFEiwApwLevZG9wEQyAMBbTisTeQngQtorGXC4t9NNWX4DA-4Hmk5WKJD_qEaMGBoCoMsQAvD_BwE<br>Anti-CD31 (1:200; AF3628; R&D SYSTEMS), https://www.rndsystems.com/products/mouse-rat-cd31-pecam-1-antibody_af3628<br>Anti aSMA (1:200; M0851; clone 1A4; DAKO), https://www.agilent.com/en/product/immunohistochemistry/antibodies-controls/primary-antibodies/actin-(smooth-muscle)-(concentrate)-76542<br>Anti-Cleaved Caspase 3 (1:200; 9661; Cell Signaling), https://www.cellsignal.com/products/primary-antibodies/cleaved-

	caspase-3-asp175-antibody/9661<br>Anti- Ki67 (1:200; PA5-19462; Thermo Fisher SCIENTIFIC), https://www.thermofisher.com/antibody/product/Ki-67-Antibody-Polyclonal/PA5-19462<br>Anti-FSP1 (1:300; 07-2274; Merck), https://www.merckmillipore.com/SE/en/product/Anti-FSP1-S100A4-Antibody,MM_NF-07-2274?ReferrerURL=https%3A%2F%2Fwww.google.com%2F<br>Anti-CAIX antibody (1.400; NB100-417; NOVUS), https://www.novusbio.com/products/carbonic-anhydrase-ix-ca9-antibody_nb100-417<br>FITC-conjugated mouse anti-pimonidazole monoclonal antibody (1:100; clone 4.3.11.3, Hypoxyprobe), https://search.cosmobio.co.jp/cosmo_search_p/search_gate2/docs/HPI_/HP2200.20140110.pdf<br>Anti-CD4 (1:50; BD Pharmingen; 550280), https://www.bdbiosciences.com/us/reagents/research/antibodies-buffers/immunology-reagents/anti-mouse-antibodies/cell-surface-antigens/purified-rat-anti-mouse-cd4-rm4-5-also-known-as-rm45/p/550280<br>Anti-CD8a (1:50; BD Pharmingen; 550281), https://www.bdbiosciences.com/us/reagents/research/antibodies-buffers/immunology-reagents/anti-mouse-antibodies/cell-surface-antigens/purified-rat-anti--mouse-cd8a-53-67/p/550281<br>Anti-Iba1 (1:200; DAKO; 019-19741), https://labchem-wako.fujifilm.com/europe/product/detail/W01W0101-1974.html<br>Alexa Fluor 555-labelled goat anti-rat (1:200; A21434; Thermo Fisher SCIENTIFIC), https://www.thermofisher.com/antibody/product/Goat-anti-Rat-IgG-H-L-Cross-Adsorbed-Secondary-Antibody-Polyclonal/A-21434<br>Alexa Fluor 647-labelled goat anti-rabbit (1:200; A-21244; Thermo Fisher SCIENTIFIC), https://www.thermofisher.com/antibody/product/Goat-anti-Rabbit-IgG-H-L-Cross-Adsorbed-Secondary-Antibody-Polyclonal/A-21244<br>Alexa Fluor 555-labelled donkey anti-goat secondary antibody (1:200; A-21432; Invitrogen), https://www.thermofisher.com/antibody/product/Donkey-anti-Goat-IgG-H-L-Cross-Adsorbed-Secondary-Antibody-Polyclonal/A-21432<br>Alexa Fluor 488-labeled donkey anti-mouse (1:400; A21202; Thermo Fisher SCIENTIFIC), https://www.thermofisher.com/antibody/product/Donkey-anti-Mouse-IgG-H-L-Highly-Cross-Adsorbed-Secondary-Antibody-Polyclonal/A-21202<br>Alexa Fluor 488-labeled donkey anti-rabbit (1:400; A21206, Thermo Fisher SCIENTIFIC), https://www.thermofisher.com/antibody/product/Donkey-anti-Rabbit-IgG-H-L-Highly-Cross-Adsorbed-Secondary-Antibody-Polyclonal/A-21206<br>Cy5-labelled goat anti-rat antibody (1:200; AP183S; Invitrogen), https://www.fishersci.se/shop/products/anti-rat-igg-h-l-cy-5-conjugated-polyclonal-thermo-scientific-novex/10308083#?keyword=AP183S%3B+Invitrogen<br><br>Flow cytometry<br>Alexa Fluor 647–conjugated anti-mouse F4/80 antibody (1:100; 123122, BioLegend), https://www.biolegend.com/en-us/products/alexa-fluor-647-anti-mouse-f4-80-antibody-4074<br>APC–conjugated anti-mouse CD3 antibody (1:100; 17-0032-80, Invitrogen), https://www.thermofisher.com/antibody/product/CD3-Antibody-clone-17A2-Monoclonal/17-0032-82<br>APC–conjugated anti-mouse CD4 antibody (1:50; 100411, BioLegend), https://www.biolegend.com/en-us/products/apc-anti-mouse-cd4-antibody-245<br>PerCP–conjugated anti-mouse CD8 antibody (1:50; 100731, BioLegend), https://www.biolegend.com/en-us/products/percp-anti-mouse-cd8a-antibody-4256<br><br>Animal treatment<br>Anti-mouse VEGF neutralizing antibody (2.5 mg kg-1; BD0801, Nanjing, China, kindly provided by the Simcere Pharmaceutical Company)<br>Anti-mouse PDGFRβ neutralizing antibody (40 mg kg-1; 2C5, ImClone Pharmaceuticals, kindly provided by Dr. Zhenping Zhu)
Validation	We use the manufactures validated antibodies and available information from their websites as shown above in the list. The optimal condition in our animal settings was confirmed in our published articles listed below.<br>Anti-mouse VEGF neutralizing antibody:<br>Proc Natl Acad Sci U S A. 2016;113(15):4158-63. doi: 10.1073<br>Proc Natl Acad Sci U S A. 2015;112(22):E2900-9. doi: 10.1073<br>Anti-mouse PDGFRβ neutralizing antibody:<br>Nat Commun. 2016;7:12152. doi: 10.1038/ncomms12152.<br>Cell Discov. 2018;4:3. doi: 10.1038/s41421-017-0002-1. eCollection 2018

Eukaryotic cell lines

Policy information about cell lines

Cell line source(s)	E0771; Purchase source is CH3 BioSystems<br>T241; Purchase source is ATCC<br>Primary cells were isolated from C57BL/6 mice.
Authentication	The cell lines were not authenticated.
Mycoplasma contamination	We confirmed that all cell lines tested negative for mycoplasma.
Commonly misidentified lines<br>(See ICLAC register)	No commonly misidentified cell lines were used.

Animals and other organisms

Policy information about studies involving animals; ARRIVE guidelines recommended for reporting animal research

Laboratory animals	The wild type C57BL / 6 mouse strain was used, and males and females aged 4 to 6 weeks were used.
Wild animals	Wild animals were not used in this study.
Field-collected samples	The study did no use any field-collected samples.
Ethics oversight	All animal studies were approved by the North Stockholm Animal Ethical Committee.

Note that full information on the approval of the study protocol must also be provided in the manuscript.

Flow Cytometry

Plots

Confirm that:

- [x] The axis labels state the marker and fluorochrome used (e.g. CD4-FITC).
- [x] The axis scales are clearly visible. Include numbers along axes only for bottom left plot of group (a 'group' is an analysis of identical markers).
- [x] All plots are contour plots with outliers or pseudocolor plots.
- [x] A numerical value for number of cells or percentage (with statistics) is provided.

Methodology

Sample preparation	Fresh tumor tissues were cut into small pieces and were incubated in a combination of 0.15% type I collagenase (Sigma-Aldrich; catalog no. C0130) and 0.15% type II collagenase (Sigma- Aldrich; catalog no. C6885) in 1% FBS PBS at 37°C for 1h. Digested tissues were diluted with PBS and filtered with a 100-micro meter cell strainer, followed by a 70 micro meter and a 40 micro meter cell strainer.
Instrument	FACSCalibur
Software	BD Cell Quest Pro<br>GraphPad<br>Excel
Cell population abundance	We analyzed the specific cell population using Flow Cytometry together with immunohistochemistry. The results between the compared groups are similar in Flow Cytometry and immunohistochemistry.
Gating strategy	The first gate was set using FSC/SSC and the debris was excluded. Single staining was performed to identify the target cell population in tumor tissues. The analysis plots were set using the specific fluorochrome stained in target cells and GFP expressed in tumor cells to exclude non -specific signals in tumor cells. This is presented in Supplementary Figure 11.

- [x] Tick this box to confirm that a figure exemplifying the gating strategy is provided in the Supplementary Information.
